# A Similarity-Weighted Informative Prior Distribution for Bayesian Multiple Regression Models

**DOI:** 10.3389/fpsyg.2021.614236

**Published:** 2021-05-11

**Authors:** Christoph König

**Affiliations:** Department of Educational Psychology, Institute of Psychology, Goethe University Frankfurt, Frankfurt, Germany

**Keywords:** informative prior distributions, prior information, heterogeneity, similarity, Bayesian multiple regression, comparability

## Abstract

Specifying accurate informative prior distributions is a question of carefully selecting studies that comprise the body of comparable background knowledge. Psychological research, however, consists of studies that are being conducted under different circumstances, with different samples and varying instruments. Thus, results of previous studies are heterogeneous, and not all available results can and should contribute equally to an informative prior distribution. This implies a necessary weighting of background information based on the similarity of the previous studies to the focal study at hand. Current approaches to account for heterogeneity by weighting informative prior distributions, such as the power prior and the meta-analytic predictive prior are either not easily accessible or incomplete. To complicate matters further, in the context of Bayesian multiple regression models there are no methods available for quantifying the similarity of a given body of background knowledge to the focal study at hand. Consequently, the purpose of this study is threefold. We first present a novel method to combine the aforementioned sources of heterogeneity in the similarity measure ω. This method is based on a combination of a propensity-score approach to assess the similarity of samples with random- and mixed-effects meta-analytic models to quantify the heterogeneity in outcomes and study characteristics. Second, we show how to use the similarity measure ωas a weight for informative prior distributions for the substantial parameters (regression coefficients) in Bayesian multiple regression models. Third, we investigate the performance and the behavior of the similarity-weighted informative prior distribution in a comprehensive simulation study, where it is compared to the normalized power prior and the meta-analytic predictive prior. The similarity measure ω and the similarity-weighted informative prior distribution as the primary results of this study provide applied researchers with means to specify accurate informative prior distributions.

## Introduction

Informative prior distributions are a crucial element of Bayesian statistics, and play a pivotal role for scientific disciplines that aim at constructing a cumulative knowledge base. Informative prior distributions are background knowledge quantified and introduced in a Bayesian analysis. Their use allows studies to build upon each other, hence to update the knowledge base of a scientific discipline continuously. This is also a central tenet of the new statistics (Cumming, 2014). Despite the increase of Bayesian statistics in various scientific disciplines over the last years, the use of informative prior distributions is still relatively rare (for instance in Psychology, see van de Schoot et al., 2017; for Educational Science see König and van de Schoot, 2018). Thus, the potential of Bayesian statistics for cumulative science is not fully realized yet.

Goldstein (2006) states that the tentative use of informative prior distributions is due to their frequently criticized subjective nature. Vanpaemel (2011) adds the lack of methods to formalize background knowledge as another reason. From an applied viewpoint, this is more severe: if the background knowledge is inaccurate, which is the case if the prior mean does not equal the population mean, parameter estimates may be biased (McNeish, 2016; Finch and Miller, 2019). Specifying accurate informative prior distributions is a question of carefully selecting studies that comprise the body of comparable background knowledge. Psychological research, however, consists of studies that are being conducted under different circumstances, with different samples and varying instruments. Thus, results of previous studies include different sources of heterogeneity, and not all available results can and should contribute equally to an informative prior distribution (Zhang et al., 2017). This implies a necessary weighting of background information based on the similarity of the previous studies to the focal study at hand. Current approaches to account for heterogeneity by weighting informative prior distributions are either not easily accessible or incomplete. For example, the power prior weighs the likelihood of the data and requires complicated intermediate steps in order to use the quantified heterogeneity properly (Ibrahim et al., 2015; Carvalho and Ibrahim, 2020). The meta-analytic predictive prior (Neuenschwander et al., 2010) is more intuitive by weighting the informative prior distribution directly, but uses heterogeneity in outcomes only. To complicate matters further, to date there are no methods available for investigating and quantifying the similarity of a given body of background knowledge to the focal study at hand. Specifying accurate informative prior distributions, however, requires an approach that quantifies all sources of heterogeneity in a body of background knowledge into a measure of similarity, and using this measure to weight the associated informative prior distribution in a direct and intuitive way.

Consequently, the purpose of this study is threefold. We first present a novel method to combine the aforementioned sources of heterogeneity in the similarity measure ω. This method is based on a combination of a propensity-score approach to assess the similarity of samples with random- and mixed-effects meta-analytic models to quantify the heterogeneity in outcomes and study characteristics (e.g., Tipton, 2014; Cheung, 2015). Second, we show how to use the novel similarity measure ω as a weight for informative prior distributions for the substantial parameters (regression coefficients) in Bayesian multiple regression models. Third, we investigate the performance and the behavior of the similarity–weighted informative prior distribution in a comprehensive simulation study, where it is compared to the normalized power prior (Carvalho and Ibrahim, 2020) and the meta-analytic predictive prior (Weber et al., 2019). The similarity measure ω and the similarity-weighted informative prior distribution as the primary results of this study provide applied researchers with means to specify accurate informative prior distributions.

The structure of this paper is as follows. First, the conceptual background of similarity is illustrated. Next, it is shown how these sources of heterogeneity can be quantified and combined in the similarity measure ω. Based on this, the similarity-weighted informative prior distribution is described. The design and results of the simulation investigating the performance and behavior of this distribution is presented next, followed by a discussion of how the similarity measure ω and the similarity-weighted informative prior distribution contribute to building confidence in and to systemizing the use of informative prior distributions in Psychological research. Please note that, in order to keep the manuscript as accessible as possible, mathematical details are kept at a minimum.

## Conceptual Background

### The Concept of Similarity

When specifying informative prior distributions, researchers are confronted with a body of background knowledge comprised of conceptual replications of studies (Schmidt, 2009). Conceptual replications focus on the general theoretical process, without copying the methods of previously conducted studies (Makel et al., 2012). Thus, the studies differ in samples, variables, and other characteristics. Without assessing their similarity to the focal study at hand, using studies for informative prior distributions might imply an unwarranted generalization; excluding studies might be too restrictive and imply that no background knowledge is available, when in truth there is. Hence, an adequate similarity measure should take into account all relevant sources of heterogeneity in research results. Consequently, the conceptual framework of the similarity measure ω follows Shadish et al. (2002), who build upon Cronbach (1982), and distinguishes between units and treatments (*UT*), outcomes (*O*), and settings (*S*) of the studies as sources for heterogeneity. More specifically, we conceptualize *UT* as samples and predictor variables, *O* as outcome variables or effect sizes, and *S* as study characteristics commonly investigated as moderators in mixed-effects meta-analytic models. Thus, we define similarity as the variability in research results due to the three sources of heterogeneity. This differentiation takes into account that heterogeneity in outcomes is not sufficient for an adequate assessment of similarity (Lin et al., 2017). The quantification of the three sources of heterogeneity is addressed next.

### Quantifying Sources of Heterogeneity

For a similarity measure to work adequately, it is pivotal that the different sources of heterogeneity can be quantified accurately with state-of-the-art methods. More specifically, the similarity measure ω is based on three components: (a) the modified generalizability index $\bar{B}$ that is based on Tipton (2014), (b) the between-study heterogeneity τ^2^ resulting from (Bayesian) random-effects meta-analytic models, and (c) $\delta_{\tau^{2}}$, the difference between the residual variance $\tau_{res}^{2}$ of (Bayesian) mixed-effects meta-analytic models and τ^2^ (for an overview see, for instance, Jak, 2015). Each individual measure quantifies important aspects of the comparability of research results.

#### Quantifying Similarity in Predictors and Samples With $\bar{B}$

The first component of the similarity measure ω is the modified generalizability index $\bar{B}$. In its original form, the generalizability index *B* is a propensity score-based measure of distributional similarity between a sample and a population (Tipton and Olsen, 2018). We modified it so that it describes the similarity between the samples of the focal study and a previously conducted study that is part of the body of available background knowledge. The generalizability index and its modified version takes values between zero and one, which indicate no and perfect similarity of the two samples, respectively. It is based on *s*(**X**), a theoretical sampling propensity score defined as *s*(**X**) = Pr(*Z* = 1|**X**), and describes the probability *Z* of an individual being in the sample of the focal study (vs. being in the sample of the previously conducted study) based on a set of covariates **X** (Tipton, 2014). The sampling propensity score can be estimated by a logistic regression model log[s(X)/1-s(X)] = α_0_+α_*m*_+X_*m*_, where *m* = 1, *m* is the number of covariates. Adapting Tipton (2014), for a set of covariates **X** and sampling propensity score *s*(**X**), the modified generalizability index is then defined as $\beta =\int \sqrt{f_{f}\left(s\right)f_{p}\left(s\right)}ds$, where *f*_*f*_(*s*) and *f*_*p*_(*s*) are the distributions of sampling propensity scores in the sample of the focal and previously conducted study, respectively. An estimator of β is provided by a discrete version of the generalizability index $B=\sum_{h}\sqrt{w_{fh}w_{ph}}$, where *h* is the number of bins and *w*_*fh*_ and *w*_*ph*_ are the proportions of the focal and previously conducted study samples, respectively (Tipton, 2014). In case of multiple previously conducted studies, the modified version of the generalizability index *B* is calculated for each comparison of the samples of the focal and previously conducted studies. It is the average of the individual indices $\bar{B}=\frac{1}{k}\sum_{k}B_{k}$, with *k* being the number of previously conducted studies. We implemented this procedure as a kernel density estimation with a Gaussian kernel and a non-parametric bandwidth selector (Moss and Tveten, 2019), so that the number of bins does not have to be chosen a priori.

#### Quantifying Heterogeneity in Outcomes With τ^2^

The second component of the similarity measure ω is the between-study heterogeneity τ^2^, which is a measure for the variance in effect sizes, such as standardized mean differences, log-odds ratios, and more recently, partial and semi-partial correlations as effect sizes for regression coefficients (Aloe and Thompson, 2013). It is the variance component of random-effects meta-analytic models, which assume that the population effect sizes are not equal across the studies. Several studies show that this assumption is usually correct: the typical between-study heterogeneity in outcomes ranges from 0.13 to 0.24 (van Erp et al., 2017; Stanley et al., 2018; Kenny and Judd, 2019). Random-effects meta-analytic models allow individual studies to have their own effect (e.g., Cheung, 2015). Let *y_k_* be the effect found in study *k*. The study-specific model is then $y_{k}=\bar{\beta }+u_{k}+\varepsilon_{k}$ where $\bar{\beta }$ is the average effect size, *u_k_* are deviations from the average effect size, ε_*k*_ is the study-specific error term and *Var*(ε_*k*_) is the known sampling variance. The variance of these deviations *Var*(*u*_*k*_) is the between-study heterogeneity τ^2^ indicating the variability of the effect sizes across the studies included in the meta-analysis. The between-study heterogeneity is strictly positive τ^2^ > 0. When τ^2^ increases, consensus in the average effect decreases. This lack of consensus in the average effect, the uncertainty quantified by τ^2^, should be represented in a weight of an informative prior distribution. However, only the meta-analytic predictive prior distribution uses τ^2^ as weight. Both the average effect and the between-study heterogeneity τ^2^ can be estimated by Maximum Likelihood, Restricted Maximum Likelihood and Bayesian estimation methods (for overviews, see Veroniki et al., 2016; Williams et al., 2018). For situations with a small number of studies, and the known problems of ML and REML estimators regarding τ^2^ in these cases, we implemented a hierarchical Bayesian random-effects meta-analytic model to estimate τ^2^ accurately.

#### Quantifying Heterogeneity in Study Characteristics with $\delta_{\tau^{2}}$

The third component of the similarity measure ω is $\delta_{\tau^{2}}$, the difference between the residual variance $\tau_{res}^{2}$ in the effect sizes, estimated by a (Bayesian) mixed-effects meta-analytic model, and their estimated between-study heterogeneity τ^2^. Mixed-effects meta-analytic models extend random-effects meta-analytic models by introducing study characteristics as potential moderators of the effects. The study-specific model is then *y*_*k*_ = β*x*_*k*_ + *u*_*k*_ + ε_*k*_, where **x**_*k*_ is a vector of predictors including a constant of one (Cheung, 2015). Under the mixed-effects meta-analytic model, the variance of the deviations *Var*(*u*_*k*_) is the residual variance $\tau_{res}^{2}$ in the effect sizes after controlling for study characteristics as moderators. If $\tau_{res}^{2}<\tau^{2}$, the study characteristics explain variance in the effect sizes. This implies that the effect sizes not only vary across studies, but also across specific study characteristics. For example, it is possible that effects found in the 1980s differ systematically from effects found in the 2010s. Thus, there is additional uncertainty in the average effect that is quantified by $\delta_{\tau^{2}}$. If $\tau_{res}^{2}\geq \tau^{2}$, the study characteristics do not explain any variance in the effect sizes, and $\delta_{\tau^{2}}$ is truncated to zero. Hence, $\delta_{\tau^{2}}>0$ if $\tau_{res}^{2}<\tau^{2}$, and *0* otherwise. Similar to the random-effects meta-analytic models, for situations with a small number of studies we implemented a hierarchical Bayesian mixed-effects meta-analytic model to estimate $\tau_{res}^{2}$ and, subsequently, calculate $\delta_{\tau^{2}}$ accurately.

### The Similarity Measure ω

The similarity measure ω integrates the three components into a single index. It is conceptually similar to the variance component of a Bayesian hierarchical model (comparable to the *a*_0_-parameter of the power prior; Ibrahim et al., 2015; Neuenschwander et al., 2009). Thus, its use as weight for informative prior distributions places certain demands on the measure, both mathematically and conceptually. First, similar to the *a*_0_-parameter of the power prior (Ibrahim et al., 2015), the similarity measure ω needs to take values between zero and one, ω ∈ [0,1]. This avoids any potential overweighting of the quantified background knowledge, compared to the information contained in the data of the focal study. Moreover, the similarity measure ω→1 as the comparability of the previously conducted studies in the body of background knowledge and the focal study increases. On the one hand, when ω = 0 the previously conducted studies and the focal study are not comparable, and no information contained in the informative prior distribution is used. On the other hand, when ω = 1, the focal study is a direct replication of the previously conducted studies in the body of background knowledge, and the information contained in the prior distribution is used fully. Second, the similarity measure ω needs to adequately reflect the inverse relation between *B*, and τ^2^ and $\delta_{\tau^{2}}$. While an increasing *B* indicates an increased comparability, increasing τ^2^ and $\delta_{\tau^{2}}$ indicate a decreasing comparability. Thus, the similarity measure needs to align the conceptual meaning of the three indices to reflect the comparability of the focal study with the study in the body of background knowledge adequately. Third, the similarity measure ω needs to be flexible in specification and discriminate strongly across the range of plausible values especially for τ^2^ and $\delta_{\tau^{2}}$, which we know to typically range between 0.13 and 0.24 (van Erp et al., 2017; Stanley et al., 2018; Kenny and Judd, 2019). This aims at conservative estimates of ω, again to avoid the informative prior distribution overwhelming the likelihood of the data of the focal study. Considering all these requirements, the similarity measure ω can be expressed formally as,

(1) $$\omega =\left(\frac{1}{1+\exp\left[10*\left(\sqrt{\tau^{2}+\;\delta_{\tau^{2}}}-0.24\right)\right]}\right)*\bar{B}$$

Thus, the similarity measure ω essentially is a logistic function of τ^2^ and $\delta_{\tau^{2}}$ with maximum value *L* = 1, midpoint ω_0_ = 0.24 and slope *s* = 10, weighted by $\bar{B}=\frac{1}{K}\sum_{k}B_{k}$, where *k* = 1…*K* is the number of previously conducted studies. The parameters of this weighted logistic function are chosen so that the resulting values of the similarity measure ω adequately reflects the characteristics of Psychological research: the midpoint is carefully chosen following van Erp et al. (2017), and the slope is chosen to discriminate adequately across the typical range of between-study heterogeneity (Stanley et al., 2018; Kenny and Judd, 2019). We assume an additive relationship between τ^2^ and $\delta_{\tau^{2}}$. Taken together, the behavior of the similarity measure is as required: ω→1 as τ^2^ and $\delta_{\tau^{2}}$ decrease and $\bar{B}$ increases. Applying equation (1) to a situation of a Bayesian multiple regression model with three predictors and ten previously conducted studies yields three parameter-specific similarity measures, which can be used to weigh an informative prior distribution.

### Applying ω – The Similarity-Weighted Informative Prior Distribution

The similarity measure ω can now be used to weight an informative prior distribution and integrate it, without any necessary intermediary calculations, in a usual Bayesian analysis. Contrary to the power prior of Ibrahim et al. (2015), who weight the likelihood of the previously conducted studies, in this case it involves raising the informative prior distribution to the power ω, *p* (θ | *D*) ∝ *p* (*D* | θ) π (θ)^ω^ where *p* (θ | *D*) is the posterior distribution of a parameter θ, *p* (*D* | θ) is the likelihood of the data, and π (βθ)^ω^ is the similarity-weighted informative prior distribution. Because this prior distribution utilizes data from previously conducted studies, it belongs to the class of evidence-based informative prior distributions (Kaplan, 2014). We illustrate the use of the similarity measure ω as weight for an informative prior distribution with an example of a simple Bayesian multiple regression with three predictors. Let **y** be a n × 1-vector of outcomes, and **X** a n × p predictor matrix, where *n* is the sample size of the focal study and *p* = 3 the number of predictors. Then,

(2) $$y\sim N\left(\beta_{0}+\text{X}\beta ,\sigma^{2}\right)$$

is the likelihood of the Bayesian multiple regression model, with β_0_ being the intercept, β a p × 1-vector of regression coefficients, and σ^2^ being the error variance. The prior specification is as follows:

(3) $$\beta_{0}\sim N\left(0,10\right)$$

(4) $$\beta \sim N{\left(\mu_{p},SE_{p}^{2}\right)}^{\omega_{p}}$$

(5) $$\sigma^{2}\sim half-Cauchy\left(0,2.5\right)$$

Both β_0_ and σ^2^ receive weakly informative prior distributions, and the hyperparameters of the informative prior distributions (means and standard deviations) for the regression coefficients β_*p*_ are the average effects μ_*p*_ and their standard errors $SE_{p}^{2}$ estimated by multiple univariate or a single multivariate random-effects meta-analysis (Cheung, 2015; Smid et al., 2020). They are weighted by the parameter-specific similarity measures ω_*p*_. Generally speaking, as ω→0 the peak around the mean of the informative prior distribution flattens, and the distribution becomes broader. A broader prior distribution carries less information about the parameter of interest; hence, the broader the distribution the lesser its informativeness.

## Simulation

We conducted a comprehensive simulation to assess the behavior of the similarity measure ω and to investigate the performance of the similarity-weighted informative prior distribution. R-code, functions, and data of the simulation are available at https://doi.org/10.17605/OSF.IO/8AEF4.

### Design

The design consisted of the following, systematically varied factors. First, the number of previously conducted studies that are part of the available body of background knowledge (*K* = 3, 5, 10). Second, the sample sizes of the previously conducted studies, indicated by the difference between the average sample sizes of these studies and the sample size of the focal study (smaller and larger △_*N*_ = −100, 100). Third, the similarity of the predictors, indicated by the differences in means of the respective distributions (i.e., their overlap) between the previously conducted studies and the focal study (from large overlap to no overlap △_μ_ = 0.25, 0.5, 1, 2, 3). Fourth, the between-study heterogeneity in the effect sizes, thus the (lack of) consensus in the background knowledge (small to large τ^2^ = 0.025, 0.05, 0.10, 0.15, 0.20, 0.35, 0.5). Moreover, we simulated one moderator variable that explained 10% of the between-study heterogeneity in the effect sizes. Thus, the simulated amount of variance in outcomes and study characteristics is $\tau^{2}+\delta_{\tau^{2}}=0.0275,0.055,0.110,0.165,0.275,0.385,0.550$. In total, the design of the simulation consisted of 210 conditions.

### Data Generation and Analysis

We applied the following procedure to generate the datasets in each condition. First, we simulated the dataset of the focal study, according to the multiple regression model in equation (2), with fixed sample size *N*_*F*_ = 200, true regression coefficients β_*F*_ = (0.5, 0.25,−0.5) and a normally distributed error $\sigma_{F}^{2}\sim N\left(0,1\right)$. Predictors in **X**_*F*_ were drawn from standard normal distributions. Next, we constructed the database of previously conducted studies, also according to the multiple regression model in equation (2) with normally distributed error $\sigma_{D}^{2}\;N\left(0,1\right)$. As a first step, the sample size for the *k*-th (*k* = 1…*K*) study of the database was drawn from a normal distribution *N*(*N*_*P*_*i*__, 25), where *N*_*P*_*i*__ = *N*_*F*_ + △_*N*_. In the second step, for the *k*-th study of the database a vector of regression coefficients β_*k*_ was drawn from a multivariate normal distribution with mean vector μ_β_*k*__ = (0.4, 0.0, 0.3), i.e., their meta-analytic means, and variance τ^2^. Compared to β_*F*_, the mean coefficients in μ_β_*k*__ represent certainty, disagreement, and contradiction in the size of the effect. Predictors in **X**_*k*_ were drawn from normal distributions *N*(μ_*N*_*P*__, 1), where μ_*N*_*P*__ = △_μ_*P*__. This procedure was repeated one hundred times in each condition, resulting in 21,000 datasets (i.e., the simulated dataset of the focal study and the databases of the previously conducted studies).

Each dataset was analyzed with a Bayesian multiple regression model with (a) non-informative priors for the regression coefficients (pooled analysis), (b) the normalized power prior (NPP), (c) the meta-analytic predictive prior (MAP), and (d) the similarity-weighted informative prior distribution (SWIP). For the non-informative model, the datasets of the focal and previously conducted studies were pooled into a single dataset. The NPP was implemented as a standard normal-inverse gamma model as described in Carvalho and Ibrahim (2020). For both the MAP and SWIP a Bayesian random-effects meta-analysis was run with the generated database of previously conducted studies to calculate the meta-analytic mean effect, its standard error, and the between-study heterogeneity τ^2^. The meta-analytic mean effect and its standard error were used as hyperparameters of the MAP and SWIP. The meta-analysis was based on Fisher’s r-to-z transformed partial correlation coefficients using the metafor-package (Viechtbauer, 2010). This follows Aloe and Thompson (2013) who introduced partial or semi-partial correlations as adequate effect sizes for regression coefficients. The specification of the MAP model and its robustification procedure followed the standard implementation of the RBesT-package outlined in Weber et al. (2019). Prior to the SWIP analysis, the modified generalizability index $\bar{B}$ for the previously conducted studies and the similarity measure ω was calculated as in equation (1). The similarity measure ω was then introduced as parameter-specific weight for the informative prior distributions for the regression coefficients as in equation (4). All models were specified with Stan and its R interface *RStan* (Stan Development Team, 2020). Four chains each of length 2,000 with 1,000 burn-in cycles were set up. Different random starting values were supplied to each chain. Convergence was assessed using the Gelman-Rubin *R*-statistic (Gelman and Rubin, 1992), where *R* < 1.02 indicated convergence. All solutions converged.

### Evaluation Criteria

To assess the behavior of the similarity measure ω we focused on its relation to $\tau^{2}+\delta_{\tau^{2}}$ and △_μ_, and its relation to the shrinkage in the parameter estimates. Therefore, we estimated linear models. Shrinkage was defined as the difference between the focal-study estimates (the true values β_*F*_) and the estimates obtained by the similarity-weighted informative prior distribution. Moreover, comparing the performance of the different prior distributions involved, for each condition, averaging the parameter estimates and their standard errors over replications, $\bar{\beta }=\frac{1}{R}\sum_{R}\beta$ and $\bar{SE_{\beta }}=\sqrt{\frac{1}{R}\sum_{R}SE_{\beta }^{2}}$, respectively. The similarity measure behaves as expected if it decreases as $\tau^{2}+\delta_{\tau^{2}}$ and △_μ_ increase. Moreover, shrinkage should increase as the similarity increases. Good performance of the different informative prior distributions is indicated by increasing shrinkage of the parameter estimates toward their meta-analytic means, as well as decreasing standard errors of the parameter estimates, depending on the degree of similarity.

## Results

### Behavior of the Similarity Measure ω

**FIGURE 1:**
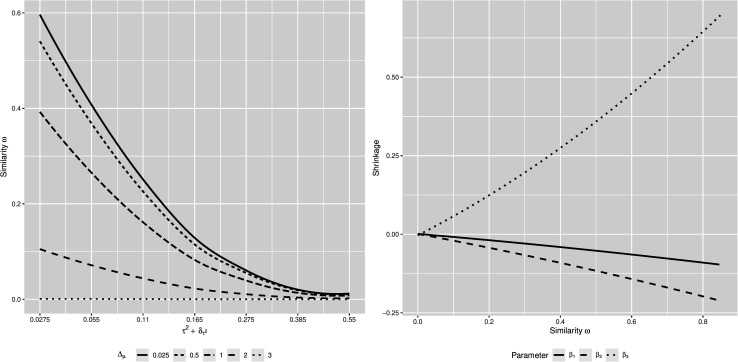
Regression curves of the relation between the similarity measure ω and $\tau^{2}+\delta_{\tau^{2}}$ for different levels of △_μ_ (**left panel**), and the relation between the shrinkage and the similarity measure ω, for each β-parameter (**right panel**), based on estimates from 21,000 simulated datasets.

Figure 1 illustrates the behavior of the similarity measure ω conditional on $\tau^{2}+\delta_{\tau^{2}}$ for different levels of △_μ_ combined for all three regression coefficients (left panel), and the behavior of the shrinkage of the estimates of the three regression coefficients, conditional on the similarity measure ω (right panel), across all simulation conditions. The similarity measure ω behaves as expected; as both $\tau^{2}+\delta_{\tau^{2}}$ and △_μ_ increase, i.e., the similarity between the focal and the previously conducted studies decreases, the similarity measure ω decreases as well. Moreover, we have a non-compensatory relation between the components of the similarity measure. High similarity in samples and predictors does not compensate for a lack of similarity regarding outcomes and study characteristics, and vice versa. The shrinkage of the parameter estimates behaves accordingly: as the focal and the previously conducted studies become more similar, indicated by an increasing similarity measure ω, the estimates of the regression coefficients shrink toward their meta-analytic means. If the focal and previously conducted studies are highly dissimilar, shrinkage is close to zero, and the estimates of the regression coefficients remain at estimates resulting from the focal study. Lastly, shrinkage is stronger when the meta-analytic means and the focal-study estimates of the regression coefficients are considerably apart (see β_3_, compared to the other two parameters). This is, however, just an effect of the distance between the values of β_3_ = −0.5 and its meta-analytic mean μ_β_3__ = 0.3. With an increasing distance between a parameter estimate and it meta-analytic mean, the potential amount of shrinkage increases as well. Moreover, the different direction of the shrinkage in case of β_3_ is due to the meta-analytic mean being larger than the focal-study estimate. In case of the other regression coefficients, their meta-analytic means are smaller than their focal-study estimates, thus the shrinkage is negative.

### Performance of the Similarity-Weighted Informative Prior Distribution

**FIGURE 2:**
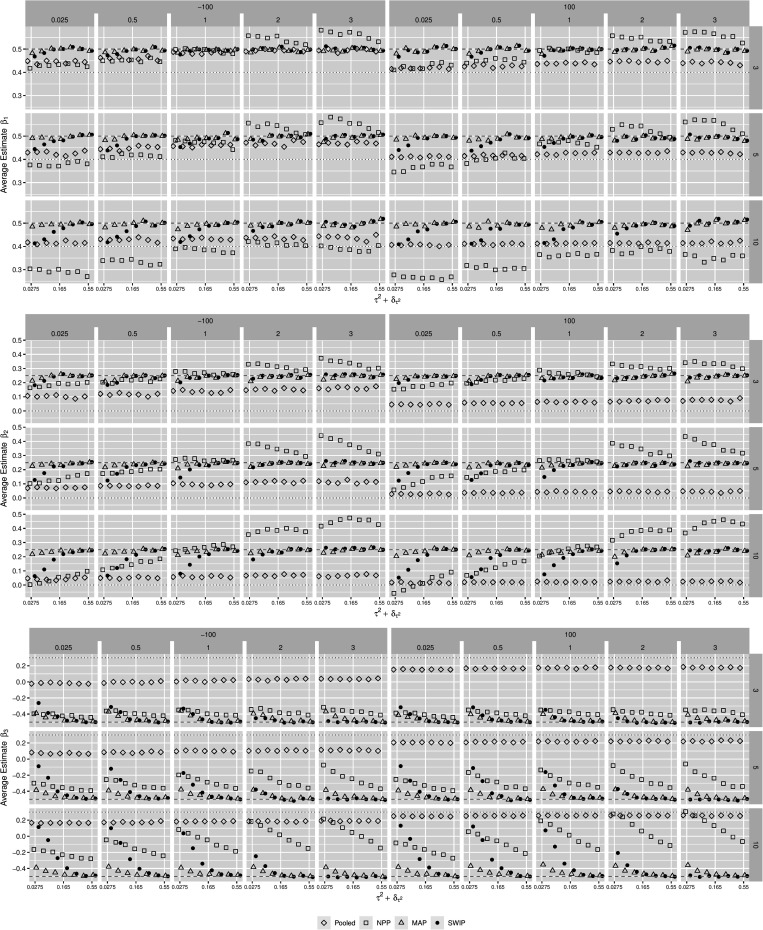
The behavior of the parameter estimates across simulation conditions. The similarity of the focal and the previously conducted studies decreases from **left** to **right**. Pooled = pooled Bayesian analysis; NPP = normalized power prior; MAP = meta-analytic predictive prior; SWIP = similarity-weighted informative prior distribution. The dashed horizontal line represents the true value of the respective regression coefficient of the focal study. The dotted horizontal line represents the true (generating) meta-analytic mean of the respective regression coefficient.

**FIGURE 3:**
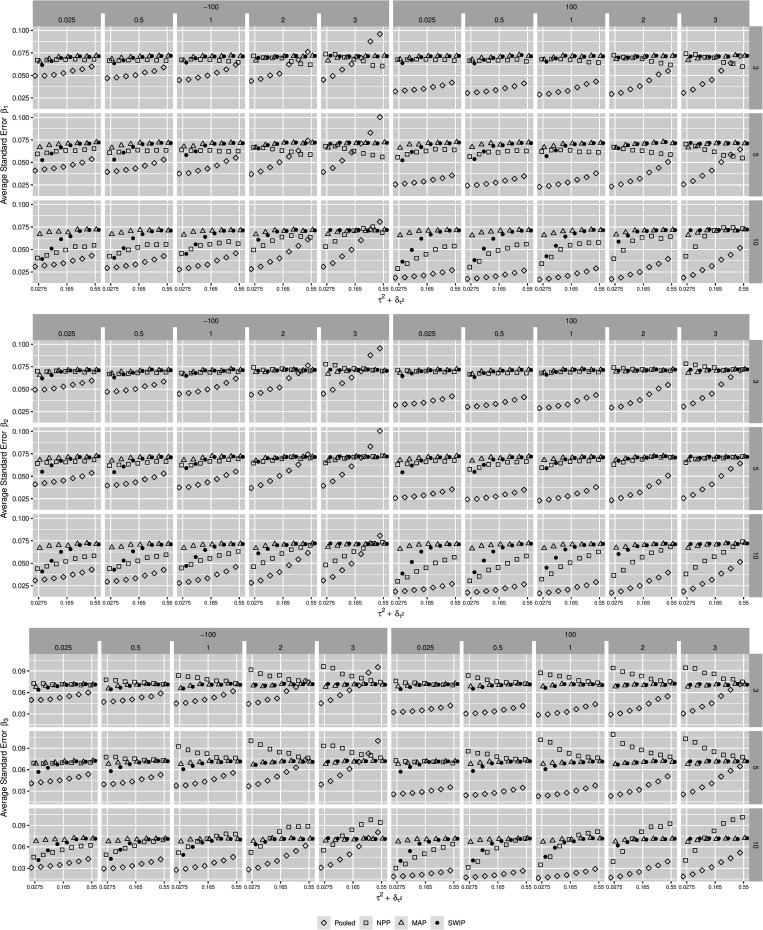
The behavior of the standard errors of the parameter estimates across simulation conditions. The similarity of the focal and the previously conducted studies decreases from **left** to **right**. Pooled = pooled Bayesian analysis; NPP = normalized power prior; MAP = meta-analytic predictive prior; SWIP = similarity-weighted informative prior distribution.

Figures 2, 3 illustrate the behavior of the estimates of the three regression coefficients and their standard errors, respectively, obtained from the pooled Bayesian analysis, the NPP, the MAP, and the SWIP, conditional on the simulated factors. The estimated regression coefficients obtained with the SWIP lie consistently between their true values β_*F*_ and their true meta-analytic means μ_β_*k*__. Shrinkage toward the true meta-analytic means is sensitive to changes in both $\tau^{2}+\delta_{\tau^{2}}$ and △_μ_. In contrast, the MAP consistently yields parameter estimates close to the true values β_*F*_, except for β_3_ when $\tau^{2}+\delta_{\tau^{2}}<.10$. Thus, the MAP is largely insensitive to changes in both $\tau^{2}+\delta_{\tau^{2}}$ and △_μ_. Compared to the NPP, shrinkage of the parameter estimates of the SWIP is comparably sensitive to changes in both $\tau^{2}+\delta_{\tau^{2}}$ and △_μ_, but more conservative. For example, when △_μ_ is large, the NPP sometimes yields overestimated parameters. Moreover, while the SWIP shrinks the parameters never beyond their estimates obtained with the pooled analysis, the NPP shrinks the parameter estimates in some cases beyond their meta-analytic means.

This general pattern is similar in case of the standard error of the parameter estimates. In case of the SWIP, the standard errors decrease as the similarity of the focal and previously conducted studies increases. More specifically, they converge to the standard errors of the pooled Bayesian analysis. This implies a similarity-dependent borrowing of information from the previously conducted studies that increases the precision of the parameter estimates of the focal study. This is true for all simulation conditions, although it is most distinct when the number of available studies is large (*K* = 10). In contrast, the standard errors of the estimates of the MAP do not converge; they largely remain at around 0.7. Thus, the MAP does not borrow information from the previously conducted studies. The standard errors of the estimates of the NPP tend to be smaller than the standard errors of the SWIP, especially when the number of previously conducted studies is large (*K* = 10). Thus, the NPP borrows more information. When the focal-study estimates and their meta-analytic means contradict (in case of β_3_), however, the standard errors of the estimates of the NPP tend to be larger, especially when the number of previously conducted studies is small and △_μ_ is large.

Overall, the performance of the SWIP is more consistent and sensitive to changes in similarity between the focal and previously conducted studies, compared to both the NPP and MAP, while yielding conservative estimates. As the similarity increases, the parameter estimates of the SWIP shrink toward the estimates of the pooled Bayesian analysis, and more information is borrowed from the body of available background knowledge. Thus, the standard errors of the parameter estimates decrease, and the estimates are more precise. In this context, the number of previously conducted studies plays a vital role. When the number is small, i.e., when there is less information to borrow, both shrinkage and precision are less distinct.

## Discussion

The purpose of this study was to illustrate a novel method to assess the similarity of studies in the context of specifying informative prior distributions for Bayesian multiple regression models. We illustrated the quantification, based on a propensity-score approach and random- and mixed-effects meta-analytic models (e.g., Tipton, 2014; Cheung, 2015), and combination of heterogeneity in samples and predictors, outcomes, and study characteristics in the novel similarity measure ω. We showed how to use the similarity measure ω as a weight for informative prior distributions for the regression coefficients, and investigated the behavior of the similarity measure ω and the similarity–weighted informative prior distribution, comparing its performance to the normalized power prior and meta-analytic predictive prior.

### The Performance of the Similarity-Weighted Informative Prior Distribution

The results of our simulation show that the parameter estimates of the similarity-weighted informative prior distribution behave similar to those of hierarchical Bayesian models: as the similarity of the focal and previously conducted studies increases, they shrink toward their pooled, meta-analytic means. Simultaneously, the precision of the parameter estimates increases because more information is borrowed from the previously conducted studies. From the perspective of cumulative knowledge creation, this behavior is desired. As evidence from comparable studies accumulates, our knowledge of the size of an effect becomes incrementally more certain until, over time, it represents the best knowledge we have (unless the evidence contradicts; Kruschke et al., 2012; König and van de Schoot, 2018). The meta-analytic predictive prior, on the one hand, does not provide this increasing certainty in the size of an effect. Compared to the similarity–weighted informative prior distribution, the similarity-dependent shrinkage is much less distinctive. Since the meta-analytic predictive prior only considers the heterogeneity in outcomes, it may be an indication that, echoing Lin et al. (2017), this is not sufficient for an adequate assessment of similarity of the focal and previously conducted studies. Parameter estimates of the normalized power prior, on the other hand, exhibit a stronger, but inconsistent shrinkage toward the pooled, meta-analytic means. From the perspective of cumulative knowledge creation, this is problematic, because the normalized power prior provides parameter estimates that are biased, and the precision of the estimates does not increase consistently as evidence accumulates.

Since the performance of the similarity-weighted informative prior distribution stands or falls with the accuracy of the components of the similarity measure ω, it is essential to estimate the random and mixed-effects meta-analytic models as unbiased as possible. This is usually based on either maximum likelihood (ML) or restricted maximum likelihood (REML) estimation (e.g., Cheung, 2015). These likelihood-based methods, however, exhibit poor performance especially when the number of previously conducted studies is small (Bender et al., 2018), additionally to the general underestimation of the between-study heterogeneity of ML-based random-effects meta-analytic models (Cheung, 2015). Several studies show a superior performance of Bayesian approaches, especially hierarchically specified random and mixed-effects meta-analytic models, in terms of the accuracy of the (residual) variance components (Williams et al., 2018; Seide et al., 2019). Thus, when using the similarity measure ω to specify the similarity-weighted informative prior distributions, we recommend using these Bayesian approaches to estimate both the mean effect size and its variance components, as illustrated in this study.

On the one hand, the similarity-weighted informative prior distribution simplifies the concept of the normalized power prior. The similarity measure is used to weight the informative prior distribution directly, which is more intuitive and less challenging than weighting the likelihood of the data from the previously conducted studies (Ibrahim et al., 2015). The complex calculation of multiple marginal likelihoods by means of bridge sampling approaches (see Carvalho and Ibrahim, 2020) is not necessary. Calculating marginal likelihoods can be complicated and time-consuming especially when the underlying models are complex (for instance, structural equation models), and their likelihood is analytically intractable (Ibrahim et al., 2015). On the other hand, the similarity-weighted informative prior distribution extends both the normalized power prior and meta-analytic predictive prior by taking into account multiple sources of heterogeneity in previously conducted studies, and quantifying these sources in the similarity measure ω. The benefits of this holistic approach are illustrated by the performance of the similarity-weighted informative prior distribution.

### Future Directions

The similarity measure ω and the similarity-weighted informative prior distribution offer various opportunities for further research. First, the inconsistent behavior of the normalized power prior may be due to the limited number of available small-sample studies (Neuenschwander et al., 2009). Thus, a limitation of this study is that we only considered sample sizes of the focal and previously conducted studies that are of a comparable order of magnitude. Investigating the performance of the similarity-weighted informative prior distribution in situations where these sample sizes differ by orders of magnitude, and where the sample sizes of the previously conducted studies vary considerably, is an important topic for further research. If the sample sizes of the focal and previously conducted studies vary considerably in size (especially when *N*_*P*_≫*N*_*F*_), it is possible to multiply the scale parameter of the informative prior distribution $SE_{p}^{2}$ by the ratio *N*_*P*_/*N*_*F*_. This can be understood as a mechanism to avoid that the prior information overwhelms the likelihood, because it flattens the distribution and makes it less informative. Second, the similarity measure can be used as the *a*_0_-parameter of the normalized power prior. Investigating the behavior of the normalized power prior in the context of a fixed–*a*_0_ approach, where the study-specific *a*_0_-parameters are fixed to the values of the study-specific similarity measures may be an interesting topic for future research. Especially because the fixed–*a*_0_ approach is considered superior to the random–*a*_0_ approach, where the comparability of the focal and previously conducted studies is inferred from the data, and the prior distribution for the *a*_0_-parameter has to be chosen carefully (Neuenschwander et al., 2009; Ibrahim et al., 2015). Third, comparing ML-based and Bayesian meta-analytic or other approaches in the context of assessing the similarity of studies, i.e., regarding their impact on the behavior of the similarity-weighted informative prior distribution, is another important topic for future studies. As mentioned above, the precision of the average effect sizes that are used as the hyperparameters of the informative prior distributions, are pivotal for the accuracy of these distributions. Identifying the correct approach, especially when the number of previously conducted studies is small (Bender et al., 2018), is crucial for the performance of the similarity-weighted informative prior distribution. Fourth, the calculation of the modified generalizability index $\bar{B}$ still requires the availability of the raw data of the previously conducted studies. This remains a limitation for the applicability of the similarity measure. Extending its applicability is a question of being able to calculate the modified generalizability index $\bar{B}$ in situations when only summary data are available. It is possible, however, to simulate a number of datasets based on correlation matrices, or means and standard deviations, and to calculate $\bar{B}$ for each of the simulated datasets. The pooled $\bar{B}$ can then be used to calculate the similarity measure. Such an approach, similar to multiple imputation or the estimation of plausible values, will be addressed and investigated in a future study. Fifth, both the similarity measure and the similarity-weighted informative prior distribution are currently only available for multiple regression models, i.e., univariate methods. It may be fruitful to extend and adapt both to multivariate methods, for example structural equation models.

### Concluding Remarks

As mentioned in the introduction to this study, specifying accurate informative prior distributions is a question of carefully selecting studies that comprise the body of comparable background knowledge. Given the considerable heterogeneity of studies that are being conducted in Psychological research (different circumstances, with different samples and instruments), the results of these studies are heterogeneous, and not all available results can and should contribute equally to an informative prior distribution. The similarity measure ω and the similarity-weighted informative prior distribution developed in this study provide researchers with tools to (a) justify the selection of studies that contribute to the informative prior distribution, and (b) to accomplish the necessary similarity-based weighting of the available background knowledge. On the one hand, the quantification of the similarity of studies, and the similarity-based weighting of prior information, are important elements of a systematization of the specification and use of informative prior distribution. Being able to justify empirically the use of previously conducted studies for the specification of informative prior distributions, on the other hand, helps building confidence in the use of informative prior distributions. The theoretical rationale of the similarity measure ω and the evidence-based nature of the similarity-weighted informative prior distribution may help to supersede the subjective notion of informative prior distributions. We hope that the similarity measure ω and the similarity-weighted informative prior distribution stimulates further research, eventually helping researchers in Psychology to move beyond non-informative prior distributions, and to finally exploit the full potential of Bayesian statistics for cumulative knowledge creation.

## Data Availability Statement

The datasets presented in this study can be found in online repositories. The names of the repository/repositories and accession number(s) can be found below: Open Science Framework—http://doi.org/10.17605/OSF.IO/8AEF4.

## Author Contributions

CK developed the conceptual background, designed, programmed, and ran the simulation, analyzed the data, and wrote the manuscript.

## Conflict of Interest

The author declares that the research was conducted in the absence of any commercial or financial relationships that could be construed as a potential conflict of interest.
